# Acknowledgement to Reviewers of *Toxics* in 2019

**DOI:** 10.3390/toxics8010005

**Published:** 2020-01-20

**Authors:** Toxics Editorial Office

**Affiliations:** MDPI, St. Alban-Anlage 66, 4052 Basel, Switzerland

The editorial team greatly appreciates the reviewers who have dedicated their considerable time and expertise to the journal’s rigorous editorial process over the past 12 months, regardless of whether the papers are finally published or not. In 2019, a total of 61 papers were published in the journal, with a median time to first decision of 20 days and a median time from submission to publication of 43 days. The editors would like to express their sincere gratitude to the following reviewers for their generous contribution in 2019:
Abete, Maria CesarinaGu, YuweiPereira, Maria De LourdesAlexiou, ChristophGuo, JingshuPerkins, EdwardAndrade, Vanda MariaHanaka, AgnieszkaPetrović, Emina KristinaAnticó, EnriquetaHartmann, ChristinaPhillips, DavidAntunes Dos Santos, AlessandraHeinbockel, ThomasPolen, TerryAvino, PasqualeHellebust, StigPulster, ErinBarrasso, RobertaHeuss-Aßbichler, SorayaQuintaneiro Antunes, Carla PatríciaBarreca, SalvatoreHiki, KyoshiroRajpurohit, SurendraBasu, AshisHill, Craig L.Raval, ManmeetBelaidi, Abdel AliHimeno, SeiichiroRenieri, Elisavet A.Bischoff, KarynHood, Darryl B.Rezania, ShahabaldinBiziuk, MarekHoriguchi, HyogoRiar, Amanjot KaurBlock, AnnaHseu, Zeng-YeiRibalta, CarlaBodin, JohannaHsiao, Chung-DerRibeiro, ClaudiaBøhn, ThomasHuang, GuannanRibeiro, EdnaBorrego, Ana AriasHudda, NeelakshiRice, Robert H.Branco, VascoIm, JeongdaeRider, CynthiaBrunelli, ElviraIslamoglu, TimurRietra, ReneBu, KaixuanJarosz-Krzemińska, ElżbietaRocio-Bautista, PriscillaBuha, AleksandraJelonek, KatarzynaRodríguez-Seijo, AndrésBus, James S.Jeong, JinyoungRomanov, VictorBusch, HaukeJohansson, Ann-ChristineRubino, Federico MariaCabral-Pinto, Marina M. S.Juneau, PhilippeRusso, Mario VincenzoCalha, IsabelKatsuta, ShoichiSabbioni, GabrieleCalliera, MauraKemp, Michael G.Sager, ManfredCao, YangKobayashi, YayoiSahu, Saura C.Caron, NathalieKosicka, KatarzynaSalanţă, Liana ClaudiaCertal, Ana CatarinaKrzyżak, JacekSamburova, VeraChan, ClementKubica, PawełSams, CraigChapman, DavidKuča, KamilSanches Silva, AnaChernyak, SergeiKudłak, BłażejSanchez-Hernandez, JuanChettle, DavidKumar, ManishSatriano, CristinaChung, Shan-ShanKyrtopoulos, SoteriosSchadt, SimoneCiulu, MarcoLadeira, CarinaSchaefer, MatthiasCohen, DanLai, Yu-HengSchöpfer, JuttaColosio, ClaudioLansman, Jeffry B.Schreinemachers, DinaCreton, StuartLau, VivianSertić, MirandaDa Costa, João PintoLavado, RamonSeth, RataneshDarvell, BrianLavkulich, Les M.Sikazwe, DonaldDe Mieri, MariaLee, Jin-YongSilke, BernardDeb, SubrataLewińska, KarolinaSiniscalco, DarioDemeilliers, ChristineLi, YingSlyvka, YuriyDeth, RichardLöytönen, MarkkuSogorb Sánchez, Miguel ÁngelDevillers, JamesLuckenbach, TillSone, HidekoDistel, MartinMacirella, RacheleStoll, RaphaelDomínguez Rebolledo, Álvaro EfrénMahmud, BasriSuliman, Hagir BDon, Aakash WelgamageMalave-Llamas, KarloSuter, Marc J.-F.Draheim, Roger R.Mancini, Francesca RomanaSzpyrka, EwaEckroat, ToddMarkowicz-Piasecka, MagdalenaSzyszkowicz, MieczyslawEdwards, JoshuaMason, HowardThevenod, FrankElnahhas, ToqaMcmorrow, TaraThoene, MichaelEric, R. GrossMedunic, GordanaTorres, Adrian GabrielErythropel, HannoMendis, HajeewakaTóth, GergőEspinoza-Derout, JorgeMikhlin, YuriUboldi, ChiaraEtxebarria, N.Miller, Gary W.Ungurianu, AncaEvans, MarleneMilosavljević, Milutin M.Valcke, MathieuEvens, AnneMitra, KaushikVance, TerrenceFantacuzzi, MarialuigiaMoretto, AngeloVelíšek, JosefFay, Michael J.Motta, Chiara MariaVesey, DavidFélix, LuisNair, VimalVidal, TâniaFernandez, Marina O.Nakai, KunihikoViitanen, Anna-KaisaFilippini, TommasoNeuberger, ManfredVillalta, PeterFu, Xiao-AnNiemann, LarsWalton, AnnMarie L.Gamet-Payrastre, LaurenceObermair, GeraldWang, PingGanapathy, VengateshOkuda, KazuhideWang, Teresa W.Gardner, Paul D.Oliver, BrianWetmore, StaceyGauthier, Patrick T.Orecchio, SantinoWickliffe, JeffreyGhosh, MonojOwsianiak, MikołajWijayawardena, AyankaGoldoni, MatteoPanderi, IreneWoelwer-Rieck, UrsulaGolmohamadi, AmirPannetier, PaulineXu, WenyanGonzález, Aridane G.Pappas, Richard StevenYao, Zhong-PingGorini, FrancescaPatyra, EwelinaZaharia, CarmenGreen, DonPeace, MichelleZeiner, MichaelaGrisoni, FrancescaPeluso, Marco E. M.Zhang, HaofeiGrollman, Arthur P.Peña-Fernández, AntonioZhu, JunjieGruszecka, AgnieszkaPenttinen, Olli-Pekka


